# Spectroscopic optical coherence tomography at 1200 nm for lipid detection

**DOI:** 10.1117/1.JBO.28.9.096002

**Published:** 2023-09-09

**Authors:** Vivek Kuttippurath, Nuria Slijkhuis, Shengnan Liu, Gijs van Soest

**Affiliations:** a Erasmus University Medical Center, Department of Cardiology, Rotterdam, The Netherlands; b Delft University of Technology, Department of Precision and Microsystems Engineering, Faculty of Mechanical Engineering, Delft, The Netherlands

**Keywords:** optical coherence tomography, spectroscopic optical coherence tomography, lipid-rich plaques, biomedical imaging

## Abstract

**Significance:**

Spectroscopic analysis of optical coherence tomography (OCT) data can yield added information about the sample’s chemical composition, along with high-resolution images. Typical commercial OCT systems operate at wavelengths that may not be optimal for identifying lipid-containing samples based on absorption features.

**Aim:**

The main aim of this study was to develop a 1200 nm spectroscopic OCT (SOCT) for the classification of lipid-based and water-based samples by extracting the lipid absorption peak at 1210 nm from the OCT data.

**Approach:**

We developed a 1200 nm OCT system and implemented a signal processing algorithm that simultaneously retrieves spectroscopic and structural information from the sample. In this study, we validated the performance of our OCT system by imaging weakly scattering phantoms with and without lipid absorption features. An orthogonal projections to latent structures-discriminant analysis (OPLS-DA) model was developed and applied to classify weakly scattering samples based on their absorption features.

**Results:**

The OCT system achieved an axial resolution of 7.2  μm and a sensitivity of 95 dB. The calibrated OPLS-DA model on weakly scattering samples with lipid and water-based absorption features correctly classified 19/20 validation samples.

**Conclusions:**

The 1200 nm SOCT system can discriminate the lipid-containing weakly scattering samples from water-based weakly scattering samples with good predictive ability.

## 1 Introduction

Many pathologies are associated with changes in tissue composition, as well as structure. Optical coherence tomography (OCT) is well suited for characterizing structural changes in optically accessible organs, providing images with ∼10  μm resolution. Tissue classification, on the other hand, in OCT images is often challenging. Qualitative feature interpretation, based on histology comparison, remains subjective and is difficult to automate. The quantitative analysis of tissue optical attenuation has been applied to the identification of tissue composition in the retina,^1^ brain tumors,^2^ prostate,^3^ and coronary artery disease.^4^^–^^7^

The attenuation coefficient is the sum of the optical scattering and absorption coefficients. Unlike the scattering coefficient, the absorption coefficient often exhibits a pronounced spectral structure that is routinely applied for the chemical analysis of biological, pharmaceutical, or industrial materials.^8^^–^^10^ As such, it has the potential to provide more chemical specificity than the scattering coefficient. Conventional image formation in OCT combines all the spectral information within its optical bandwidth to achieve optimal resolution and image quality, losing any spectral information.

In the setting of imaging coronary artery disease, tissue composition is an important factor in assessing the risk of coronary syndromes. Particularly, lipid-rich plaques detected by near-infrared spectroscopy have been associated with the occurrence of coronary events.^11^^,^^12^ A unique OCT-based lipid signature could support composition-based plaque risk assessments.

A functional extension of OCT called spectroscopic OCT (SOCT) has emerged with potential for depth-resolved spectroscopic analysis of tissues along with the high-resolution OCT images.^13^^,^^14^ This combined analysis of spectral and spatial information in OCT data is a promising method for diagnosing many diseases and identifying tissue types.^15^^–^^18^

Spectroscopic analysis in OCT can be accomplished in two different methods. The first is a hardware-based method that uses dual-wavelength band sources, and the resulting images are compared in a differential color encoded image.^19^ The second, more common way is a post-processing technique that uses time-frequency analysis of OCT data either in a frequency domain^20^^,^^21^ or spatial domain to generate depth-resolved spectra.^16^^,^^22^ Recent investigations have focused on the postprocessing-based SOCT for the characterization of lipid-rich plaque in tissues and phantoms using the absorption features of lipids.^23^^,^^24^ However, the data were acquired using OCT systems operated at a center wavelength of 1310 nm, where the lipid absorption coefficient is relatively small and lacks clearly discerning structures. This demands much reference data and training or a representative metric to visualize the property of the spectrum.

Against this background, we propose a swept laser source-based SOCT operating in a 1220 nm window where the contrast between lipid and normal tissue is more evident due to the stronger lipid absorption compared to the 1300 nm window. In this paper, we demonstrate the development of a 1.2  μm wavelength swept laser source OCT system and the signal processing approach for the simultaneous imaging and spectroscopic analysis of samples. We have validated the proposed method by imaging weakly scattering phantoms with and without the absorption features of lipids. Furthermore, we developed a predictive model to classify the lipid-containing samples and validated its performance.

## 2 Materials and Methods

### 2.1 OCT System Description

**Fig. 1:**
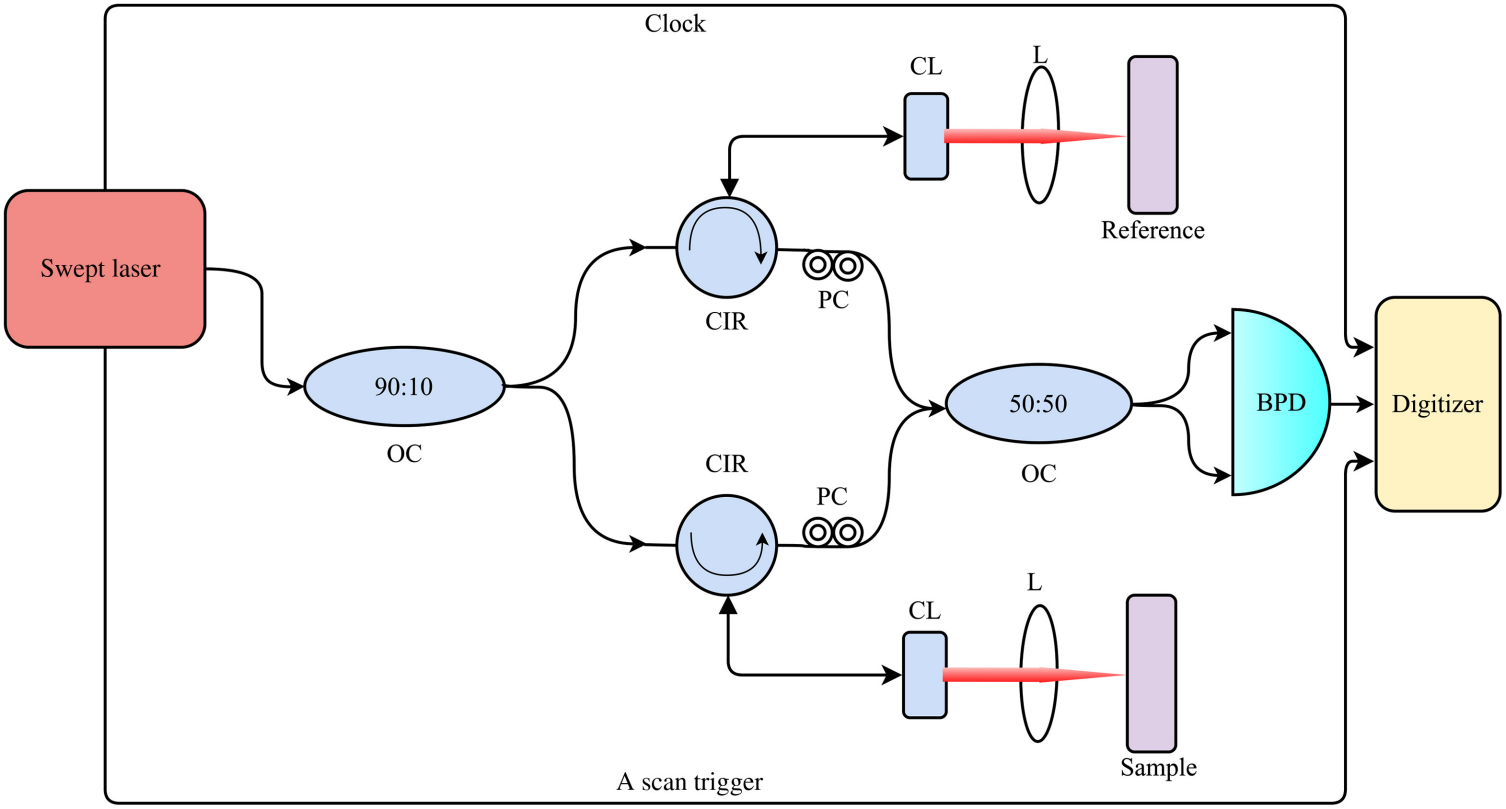
Schematic of OCT system. OC, optical coupler; CIR, circulator; CL, collimator; L, focusing lenses; PC, polarization controller; and BPD, balanced photodetector.

Figure 1 shows the schematic of the 1.2  μm swept laser source OCT system. The system used a swept laser source (Axsun Technologies Inc.) of 100 kHz sweep rate with a duty cycle of 41.9%. The laser had a spectral bandwidth of 101 nm at a center wavelength of 1220 nm and it delivers an average output power of 30 mW. In the OCT system, we used standard fiber optic components with a center wavelength of 1310 nm since commercial fiber optic components are not available specifically for the 1200 nm window. The output from the laser source was fed through a 90:10 fiber coupler (Thorlabs, TW1300R2A2) guides 90% of light toward the circulator (Thorlabs, CIR1310-50-APC) in the sample arm and 10% of light toward the circulator in the reference arm of the interferometer. The light from the fiber circulators is collimated through a fiber collimator (Thorlabs, F280APC-C). Achromatic doublets (Thorlabs, AC254-050-C) with a focal length of 50 mm are used on both arms, resulting in a lateral resolution of 25  μm at the focal plane. The system achieves an average optical power of 16.5 mW in the sample arm. The reflected light from the sample and reference mirror travels back to the respective circulators, where it is guided toward a wideband 50:50 coupler (Thorlabs, TW1300R5A2). The interference signal from the 50:50 coupler is detected by a dual-balanced photodetector (Thorlabs, PDB481C-AC). The signal from the photodetector, and the sampling clock signal generated from the laser, were digitized with a 2.5  GS/s, 12-bit digitizer (Teledyne SP Devices, ADQ32). Data acquisition was controlled using custom Python software, and the acquisition was triggered with the sweep trigger from the laser source.

### 2.2 Weakly Scattering Phantom Preparation

**Fig. 2:**
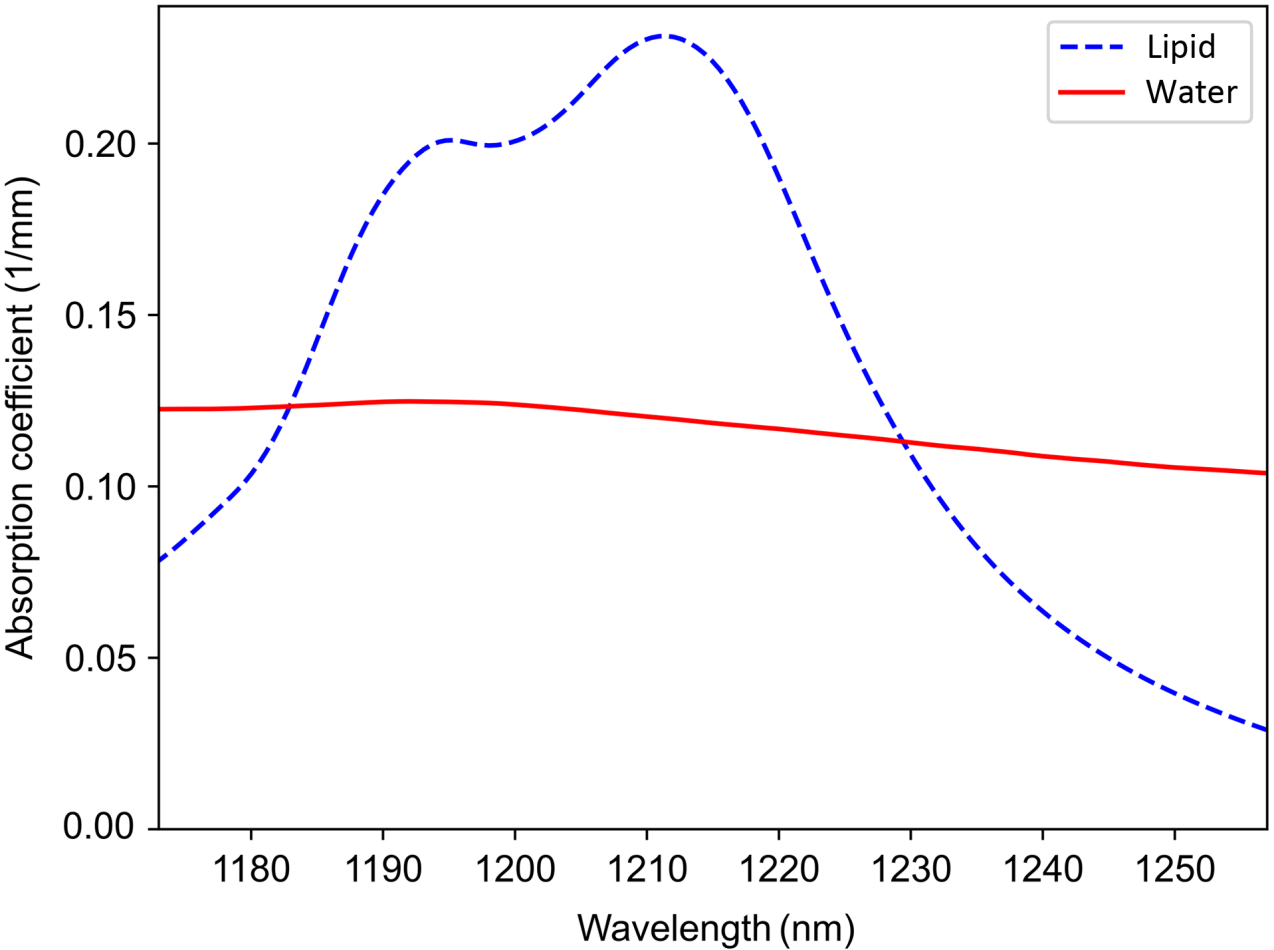
Absorption spectra of lipid-containing mineral oil and water within the spectral bandwidth of the OCT system.

The absorption spectra of mineral oil, which contains lipids and pure water obtained from literature, are shown in Fig. 2.^25^^,^^26^ As shown in Fig. 2, within the spectral bandwidth of the OCT system, lipid has a sharp absorption peak at 1210 nm, whereas the water has an almost flat absorption spectrum with a smaller absorption coefficient.

To validate our method, we fabricated two types of weakly scattering optical phantoms, which differ in their absorption features at the wavelength range of our OCT system. The first set of phantoms was intended to mimic lipid-rich tissues. We chose gel wax as the matrix material typically composed of mineral oil and polymer resin. These phantoms were prepared following a previously published recipe.^27^ The gel wax itself is optically clear. We added titanium dioxide (${\mathrm{TiO}}_{2}$) powder (Dupont™ Ti-Pure^®^ CAS No: 13463-67-7) as optical scatterers in the native gel wax (Mindsets online, FF1003). In this study, phantoms with various scattering coefficients were prepared by varying the concentrations of ${\mathrm{TiO}}_{2}$ particles from 0.05% to 0.2% w/w.

The second set of phantoms was designed with agar as a matrix material. The absorption of this material is mainly due to water in the wavelength range of our OCT system. We employed the same concentrations of ${\mathrm{TiO}}_{2}$ powder in the agar matrix, to approximate the scattering coefficients of the gel wax phantoms. The background scattering of the agar is assumed to be smaller than that of the added ${\mathrm{TiO}}_{2}$. The samples were prepared by dissolving 2% agar (Sigma-Aldrich, A6549) and varying concentrations of ${\mathrm{TiO}}_{2}$ powder in 100 ml of deionized water. The solution was heated to 94°C and continuously stirred until the agar and ${\mathrm{TiO}}_{2}$ particles were wholly dissolved in water. The prepared solution was then allowed to cool down to room temperature and stored in a refrigerator for 24 h. In our phantom study, the samples described above were prepared in rectangular shapes, and two-dimensional B-scan imaging was performed with 1000 A-scans in a frame.

Finally, independent lipid and non-lipid phantoms were used for validation. A gel wax phantom and a weakly scattering polyvinyl alcohol (PVA) phantom, both with 0.1% ${\mathrm{TiO}}_{2}$ scatterer concentration were used. PVA (5% w/w) and ${\mathrm{TiO}}_{2}$ were mixed in water and heated to 70°C and left to cool. The solution underwent three freeze-thaw cycles, creating an aqueous scattering gel.

### 2.3 OCT Signal Processing and Image Reconstruction

**Fig. 3:**
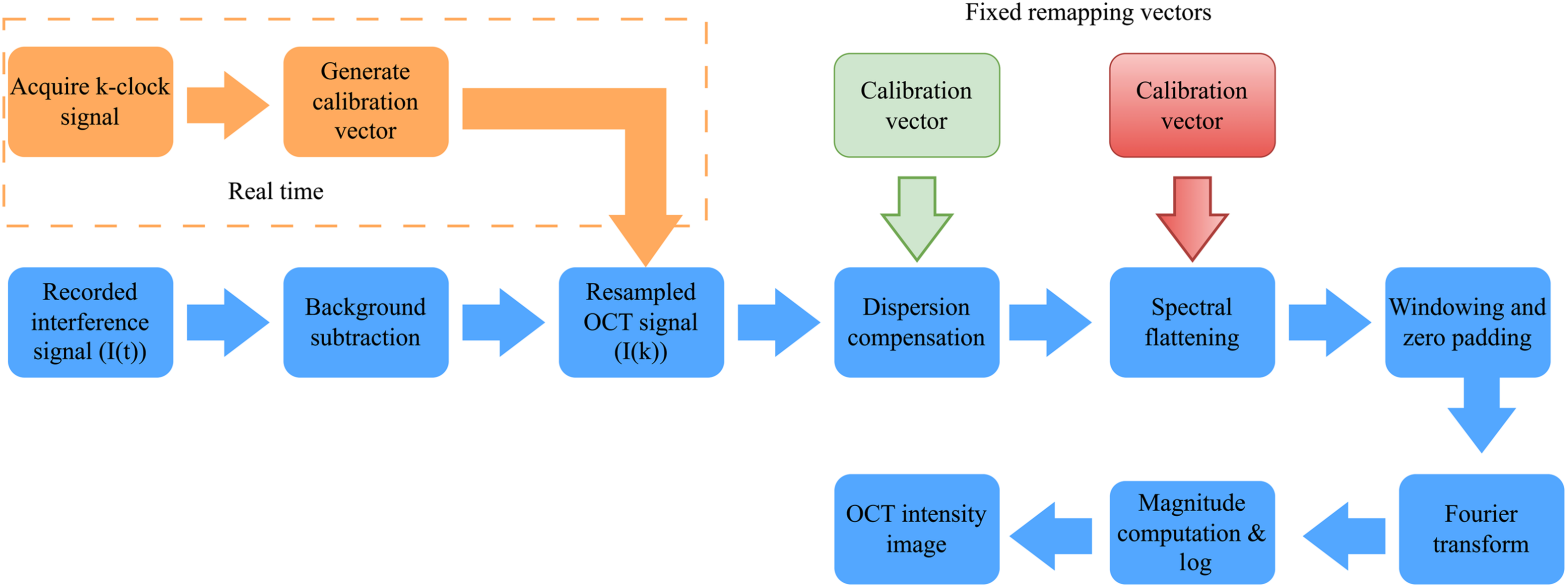
Block diagram of the signal processing algorithm to generate OCT intensity image.

Figure 3 shows the block diagram of signal processing steps to reconstruct the OCT intensity image. First, the background signal is measured by blocking the OCT system’s sample arm and subtracting it from the acquired interferogram. The dual-balanced photodetection in the system is not perfect, particularly due to imperfect power balancing over the whole spectral range. Therefore, residual excess noise remains in the system. Moreover, any imperfection in background subtraction results in a DC component in the signal. Therefore, the background subtracted interference signal is further filtered using a digital bandpass filter. The smaller and larger frequency components of the acquired interferogram that corresponds to the signals from the surface and highest imaging depth of the sample are used as the cut-off frequencies of the filter. These frequency components are determined by the Fourier transform of the acquired interferogram. The swept laser source used in our OCT system has nonlinear sweep characteristics in the wave number (k) domain, requiring k-linear resampling of the acquired interference signals. We employed a numerical resampling method that involves linearizing the unwrapped phase of the sampling clock signal to generate the calibration vector for interpolation as presented by Wu et al.^28^

Once k-linearized, the interference signals were processed for dispersion compensation. We adopted a method^29^ to numerically compensate the dispersion mismatch between the interferometer’s sample and reference arm optics. To determine the system dispersion mismatch, 200 A-scan measurements of a mirror at a fixed location on the sample arm were carried out. After background subtraction and k-linearization, the interferograms were averaged, and the Hilbert transform was applied to determine the spectral phase. A linear fit on the unwrapped Hilbert phase was computed. Then, the nonlinear phase $\left[\phi_{\mathrm{disp}}(k)\right]$ due to system dispersion mismatch was estimated by subtracting the spectral phase and the linear fit. The estimated nonlinear phase $\left[\phi_{\mathrm{disp}}(k)\right]$ was stored as a calibration vector in computer memory and it was employed to compensate the dispersion mismatch by multiplying the k-linearized interference signals with exp $\left[-i\phi_{\mathrm{disp}}(k)\right]$.

In our OCT system, the optical spectrum at the interferometer’s output significantly deviates from the original laser spectrum since fiber optic components used in the system are not optimized for the 1200 nm wavelength window. This could effectively reduce the theoretically attainable OCT axial resolution. Therefore, in the signal processing chain, we implemented an additional signal processing technique of spectral flattening.^30^ This signal processing step compensates for the spectrum shape change due to wavelength-dependent attenuation of fiber optic components in the system. Moreover, we implemented a multiwindow signal processing using a series of Kaiser–Bessel windows on the spectral flattened interference signal.^30^ This effectively reduces the axial side lobe level and achieves a better axial resolution than convention signal processing windows. The interference signals were zero-padded to four times their original length before the Fourier transform. The logarithmic compressed OCT intensity image was generated using the magnitude of the complex signal in the spatial domain.

### 2.4 Spectroscopic OCT Data Processing

#### 2.4.1 Attenuation spectral analysis

The OCT backscattered intensity signal [I(z)] from depth z of the sample can be modeled using the single scattering model that obeys Beer-Lambert law as

⟨I(z)⟩=I0T(z)S^(z)e−μtz, (1)

where T(z) is the longitudinal point spread function of single mode fiber-based OCT system, S^(z) represents the signal-roll off of the system, and $\mu_{t}$ is the total attenuation coefficient of the sample. The attenuation coefficient $\mu_{t}$ of a sample has both absorption $\mu_{a}$ and scattering ($\mu_{s}$) contributions ($\mu_{t}=\mu_{a}+\mu_{s}$).

Since the scattering and absorption coefficients of a material are wavelength-dependent parameters, the measurement of spectral-dependent OCT intensity profiles [I(λ,z)] can offer material classification based on either scattering or absorption features.^23^^,^^31^^,^^32^ In this study, we exploited the distinct absorption features of water and lipid containing samples for the classification of materials.

**Fig. 4:**
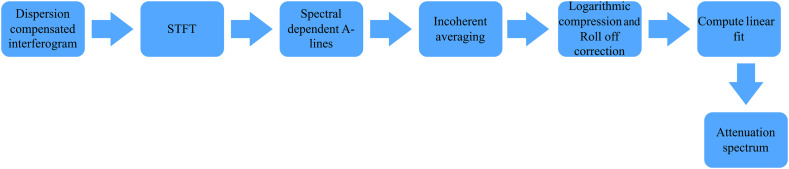
Block diagram of signal processing algorithm to generate attenuation spectrum.

A schematic overview of SOCT data processing is shown in Fig. 4. First, the k-linearized and dispersion compensated interference signal of the sample were analyzed with time-frequency distributions. In our study, we used the short time Fourier transform with Hanning windows for the time-frequency analysis, which resulted in a spectral and spatial resolution of 8.4 nm and 101  μm, respectively. To reduce the speckle noise in the spectral-dependent OCT intensity profiles, we averaged the spectrograms obtained from 200 A-scans, which were sampled by 100 lateral speckles over the scanning range of 2.5 mm. The scanning range in one B scan was 35 mm, limited by the length of the phantom samples. Subsequently, the averaged spectral-dependent intensity profiles were divided by the longitudinal point spread function of the OCT system and then linearized by logarithmic compression.^33^ We also included the correction for sensitivity roll-off of the OCT system. Then, we extracted the wavelength-dependent attenuation coefficients $\left[\mu_{t}(\lambda )\right]$ by fitting straight lines on the logarithmic compressed spectral-dependent intensity profiles. The resulting attenuation coefficients $\mu_{t}(\lambda )$ from different lateral positions on the sample were further processed for material classification.

#### 2.4.2 Extraction of absorption coefficients

To extract the absorption spectrum from the sets of weakly scattering and absorbing samples, we formulated a linear equation system using the measured scatterer concentration-dependent attenuation spectra $\mu_{t,i}(\lambda )$ for a range of known concentrations of ${\mathrm{TiO}}_{2}$ scatterers in the phantoms. The N linear equation system with two unknown parameters for each wavelength can be written as

$$\mu_{t,i}(\lambda )=c_{i}\sigma_{{\mathrm{TiO}}_{2}}+\mu_{a}(\lambda ),$$ (2)

where $c_{i}$ is the concentration of scatterers in the sample i, and $\sigma_{{\mathrm{TiO}}_{2}}$ is the scattering cross-section of the ${\mathrm{TiO}}_{2}$ particles. The linear equation system for each wavelength can be solved by fitting the measured scattering concentration-dependent attenuation coefficients $\mu_{t,i}(\lambda )$ to a line of the form y=mx+b, using linear least square method and the unknown parameter of wavelength dependent absorption coefficients $\mu_{a}(\lambda )$ can be obtained as the intercept (b). Here, the scattering cross section is constant over the operating wavelength of the laser source and is obtained as the slope of straight line (m).

#### 2.4.3 Classification of samples using a predictive model

We performed multivariate analysis to classify the materials based on their absorption features. For this analysis, a training dataset to build the predictive model was generated by measuring the attenuation spectra from ten different locations of each sample. An orthogonal projections to latent structures-discriminant analysis (OPLS-DA) model^34^ was fitted in SIMCA 17 (Umetrics, Sweden), comparing the mean attenuation spectra of lipid-containing gel wax to the mean spectra of water-based agar phantoms. In this analysis, the retrieved spectral absorption coefficients were defined as the model’s variables, and the sample type (gel wax or agar) was defined as observations. The quality of fit was reported as $R^{2}$ and the predictability of the model was reported as $Q^{2}$. The model was considered significant when $R^{2}>0.5$ and $Q^{2}>0.5$ and was further validated with a permutation test and by a sevenfold cross-validation analysis of variance (CV-ANOVA).^35^

To comprehend the advantages of classification of samples based on attenuation spectra, we also performed multivariate analysis based on normal attenuation coefficient ($\mu_{t}$) of samples. The normal attenuation coefficients of the samples were obtained from the OCT intensity profiles.^6^ For this study, the training and validation dataset were generated by measuring the attenuation coefficients from an appropriately averaged OCT intensity profiles from different locations of each sample.

## 3 Results

### 3.1 Swept-Source OCT System Performance

**Fig. 5:**
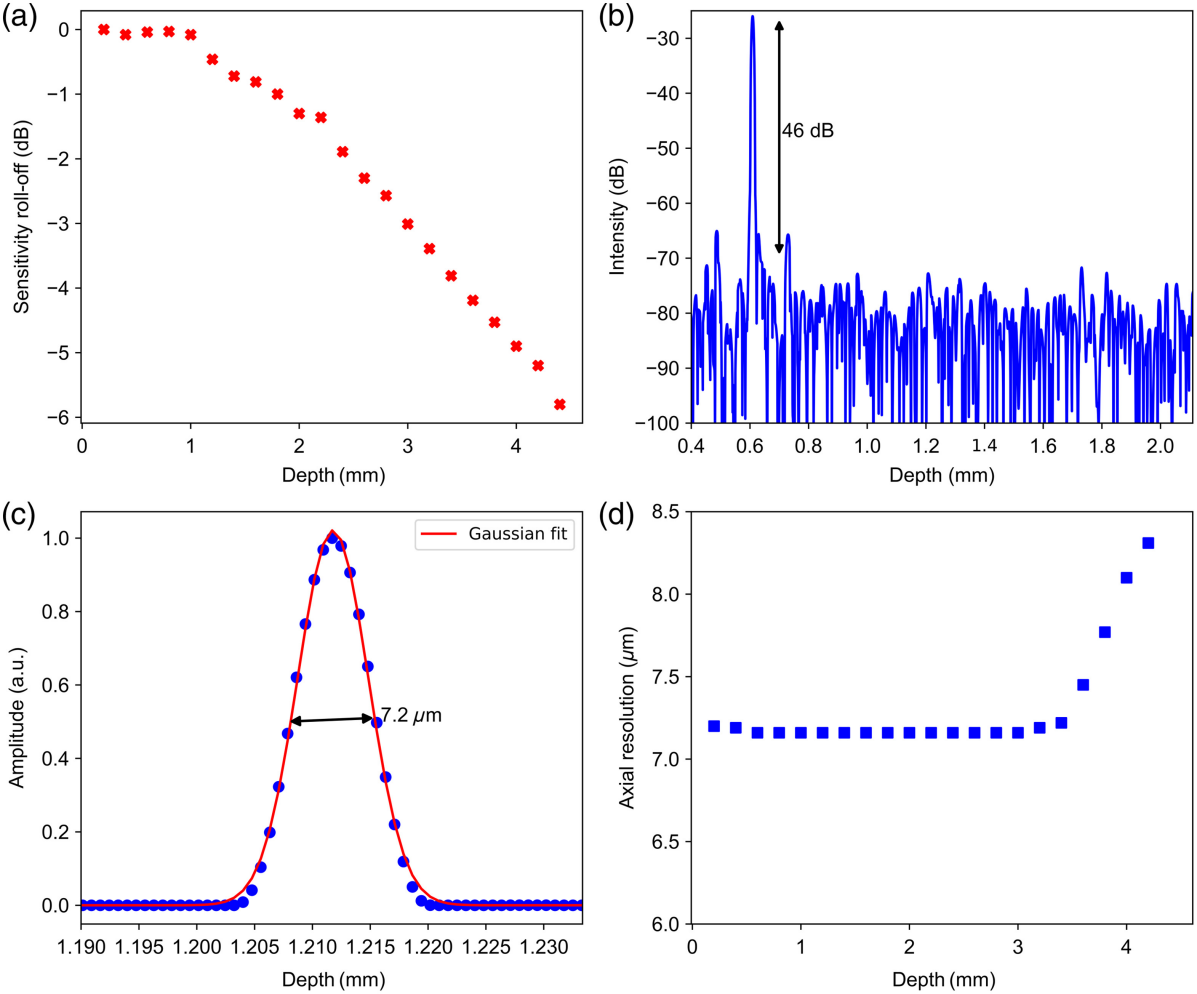
(a) Sensitivity roll-off performance of OCT system. (b) Dynamic range of OCT system. (c) Point spread function of OCT system. (d) Axial resolution of the OCT system at different imaging depths.

To assess the performance of the OCT system over the entire imaging range, a series of measurements were performed by positioning a silver coated mirror at the focal plane of the sample arm along with a Neutral Density filter of −32  dB attenuation. The sensitivity was measured as a function of depth by varying optical path length in the reference arm of the interferometer. The maximum sensitivity of the system was experimentally determined as 95 dB. The measured maximum sensitivity is ∼20  dB lower than the shot noise limited value. This larger deviation of sensitivity from shot noise limited value could be attributed to two reasons. One is the excess noise of swept laser source used in the system, and the additional losses and noise due to guidance of multiple spatial modes in standard optical fiber components as the laser used in the system operates below the cut-off wavelength of SMF-28. Therefore, the sensitivity of the system can be improved in future using components made from optical fibers with lower cut-off wavelength than the minimum wavelength of the laser source used in the system. Figures 5(a) and 5(b) display sensitivity roll-off performance as a function of the imaging depth and dynamic range of our OCT system. The system has a 6 dB sensitivity roll-off at 4.2 mm in air and the dynamic range of 46 dB. The axial resolution of the OCT system was measured as the full-width half maximum of the OCT A-scan profile of a perfect reflector. Figure 5(c) shows the measured axial point spread function and the Gaussian fit at the axial distance of 0.81 mm. The measured axial resolution was 7.2  μm in air, close to the theoretical axial resolution of 6.5  μm. The achieved axial resolution is 1.38 times narrower than the commercially available 1310 nm OCT system.^36^ The depth dependence of the axial resolution is shown in Fig. 5(d). The axial resolution preserves the value around 7.2  μm up to a depth of 3.3 mm, and it slowly increases to 8.6  μm at a higher imaging depth. This slight variation in axial resolution at higher imaging depth could be due to the calibration error in the k-linearization algorithm and mismatch in dispersion at higher imaging depth.

### 3.2 OCT Intensity Imaging and Spectroscopic Analysis

**Fig. 6:**
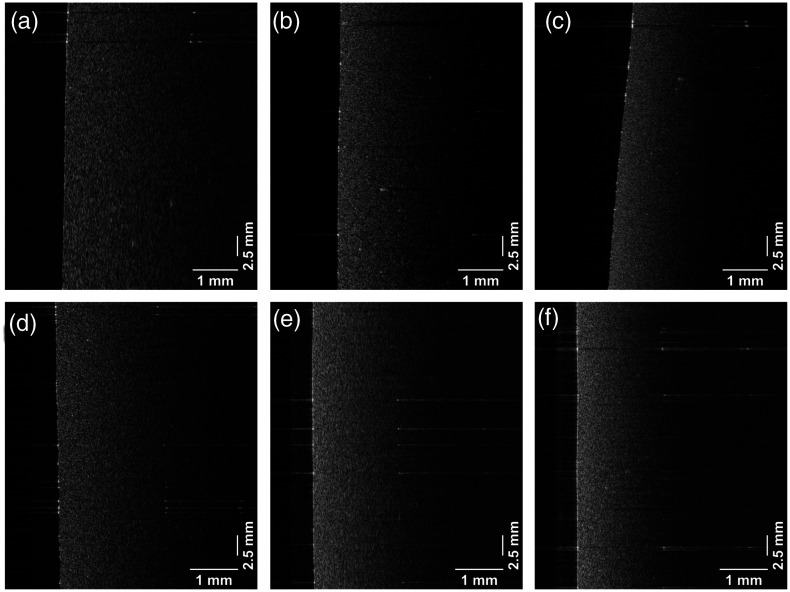
OCT images of phantoms. (a)–(c) Gel wax with 0.05% TiO2, 0.1% TiO2, and 0.2%, respectively. (d)–(f) Agar phantoms with 0.05%, 0.1%, and 0.2% TiO2 scatterers.

The OCT B-scan intensity images were generated for each phantom with different scatterer concentrations, as shown in Fig. 6.

**Fig. 7:**
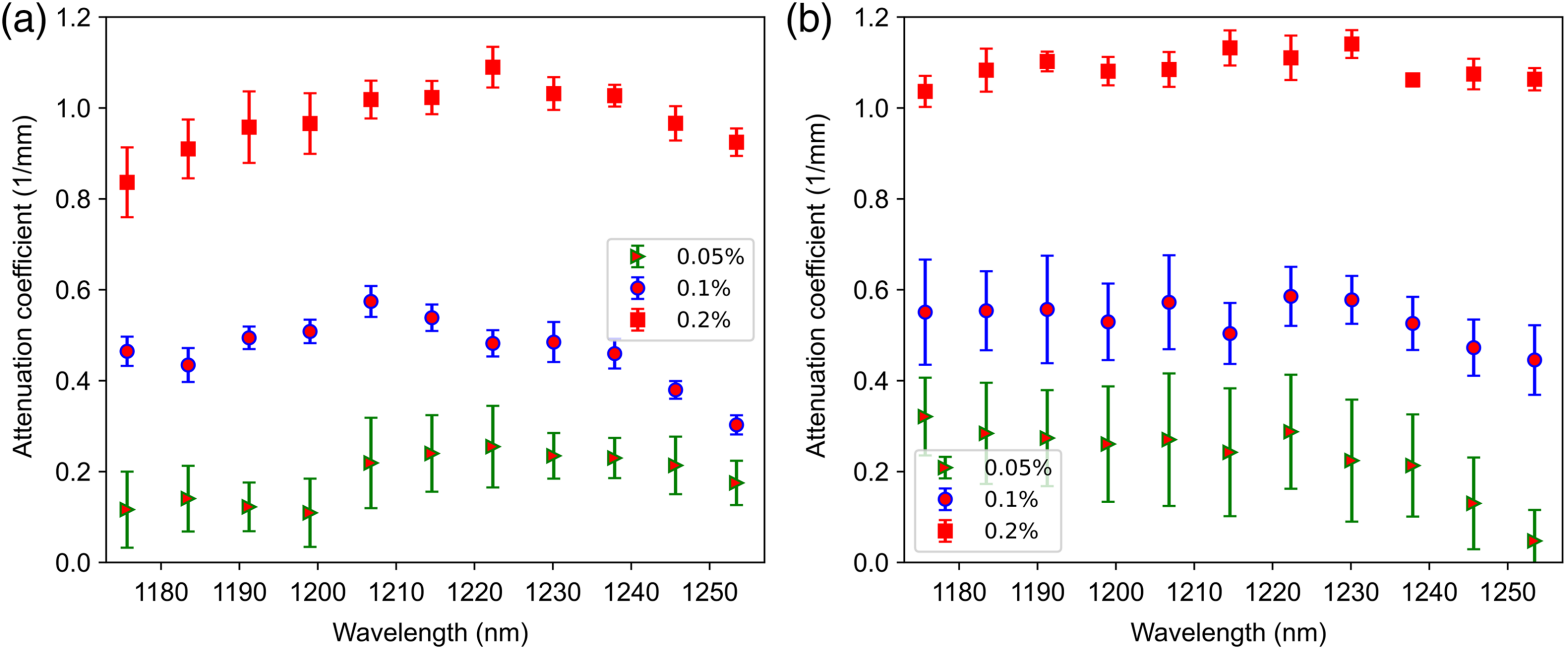
Optical attenuation spectra of (a) gel wax phantoms and (b) agar phantoms with 0.05%, 0.1%, and 0.2% TiO2 scatterers.

Figures 7(a) and 7(b) show the measured attenuation spectra $\left[\mu_{t}(\lambda )\right]$ of the gel wax and agar phantoms from an averaged spectrogram over the scanning range of 2.5 mm. The error bars in attenuation spectra represents the variation of measured spectral dependent attenuation coefficients in terms of root mean square error (RMSE). The RMSE is calculated from the residuals of linear fit on the averaged spectral dependent intensity profiles over the scanning range of 2.5 mm. The measured attenuation spectra are analyzed with respect to the absorption feature of the phantom materials. The absorption signature of lipid in the gel-wax phantoms is correlated with a small peak around 1210 nm in the attenuation spectrum. On the other hand, the agar phantoms exhibit a flat attenuation spectrum, which is associated with the absorption spectrum of water in the phantoms. The absorption behavior of phantom materials in the attenuation spectra is discernable only at the lower concentration of scatterers, whereas the measured spectra significantly differed at higher scatterer concentrations. This significant deviation of spectral shape with increasing concentration of ${\mathrm{TiO}}_{2}$ is attributed to the dominant contribution of light scattering with minimal absorption to the overall attenuation coefficient. The measured values at the edges of the spectrum were considered erroneous due to the lower signal-to-noise ratio of the spectrum at the ends.

The measured attenuation coefficient at each wavelength increases linearly with the concentration of ${\mathrm{TiO}}_{2}$ scatterers. This confirms that the wavelength-dependent absorption coefficients can be obtained by solving the linear equation system established for different scattering concentrations.

**Fig. 8:**
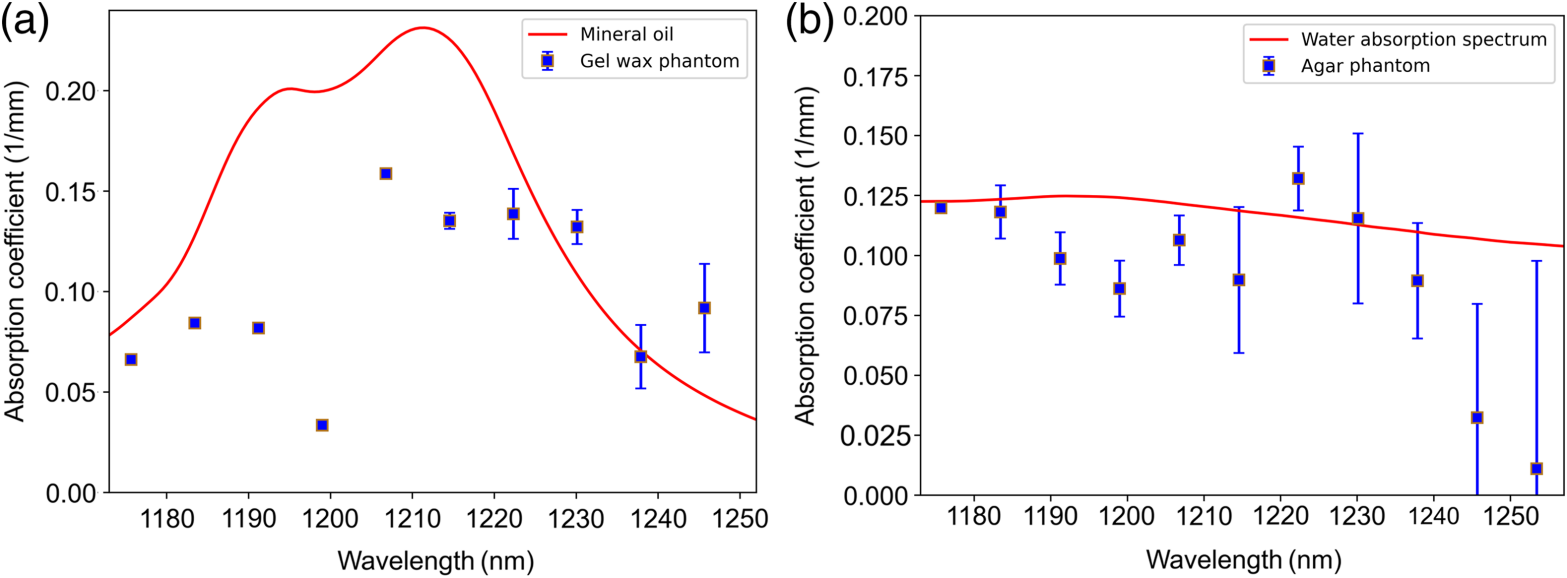
(a) Retrieved absorption spectrum of gel wax phantom and reference spectrum of mineral oil. (b) Retrieved absorption spectrum of agar phantom and the reference spectrum of water.

Figures 8(a) and 8(b) show the reference absorption spectra of mineral oil and water along with the absorption spectra of gel wax and agar phantoms that we derived by solving Eq. (2) using the measured attenuation spectra for different scattering concentrations. The estimated absorption spectrum of gel wax has a prominent absorption peak at 1210 nm with an absorption coefficient of $0.15\;{\mathrm{mm}}^{-1}$, which is close to those published in the previous studies. Error bars in Fig. 8 represent the variation of estimated absorption coefficients in terms of root means square error. On the other hand, the agar phantom demonstrates a broad absorption spectrum similar to that of water in the measured wavelength range with an estimated absorption coefficient of $0.12\;{\mathrm{mm}}^{-1}$ at 1200 nm. This result confirms the evidence of absorption features in the attenuation spectra, so a predictive model can be used to classify the lipid and non-lipid weakly scattering samples.

**Fig. 9:**
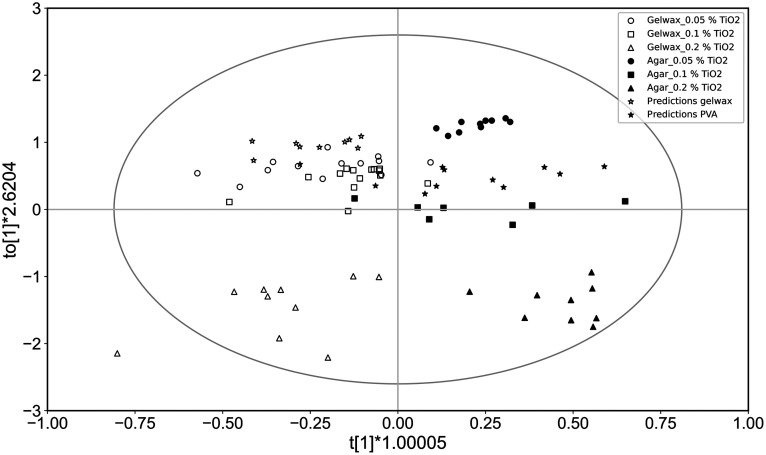
OPLS-DA score plot of gel wax phantoms (open symbols) and agar phantoms (solid symbols) as training set and gel wax phantoms (open stars) and PVA phantoms (solid stars) as validation set. The horizontal axis shows variation between groups, and the vertical axis shows variation within groups.

Figure 9 shows the outcome of the predictive model. The open and solid symbols of circles, squares, and triangles represent the lipid-containing gel wax and the water-based agar phantoms at 0.05%, 0.1%, and 0.2% scatterer concentrations, respectively. This predictive model shows the significant quality of fit $\left[R^{2}(X)\right]$ of 0.653 with a predictive ability ($Q^{2}$) of 0.591. The sevenfold CV-ANOVA of model results in the $p=2.{158e}^{-09}$, which is considerably smaller than the significance threshold.

In Fig. 9, the star symbols represent the validation set of samples results from a fit to the entire training dataset. The open symbols of stars indicate the validation set of lipids containing gel wax samples, and the solid stars stay for water-based PVA samples. In the validation dataset, 95% of the datasets were correctly assigned to their respective classification.

**Fig. 10:**
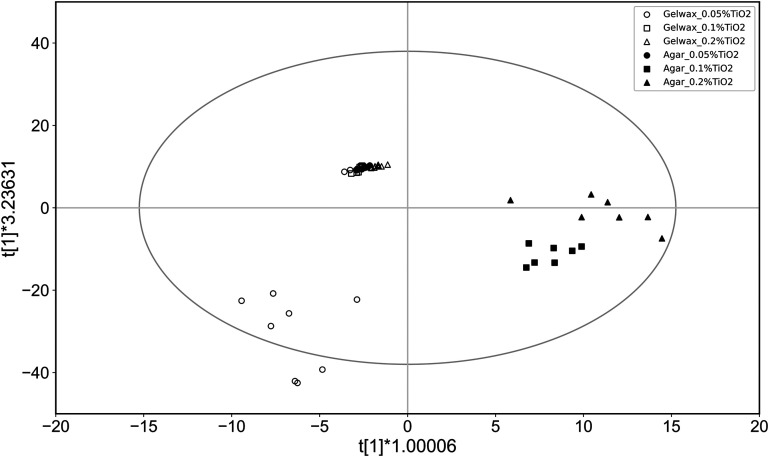
OPLS-DA score plot based on normal attenuation coefficients of gel wax (open symbols) and agar phantoms (solid symbols). The horizontal axis shows variation between groups, and the vertical axis shows variation within groups.

Figure 10 shows the OPLS-DA model based on normal attenuation coefficients of gel wax and agar phantoms. The model based on attenuation coefficients shows quality of fit $\left[R^{2}(X)\right]$ of 0.468 with a predictive ability ($Q^{2}$) of 0.446. The value of $R^{2}<0.5$ indicates a poor quality of fit and hence the classification based on attenuation coefficients is insignificant. Consequently, the agar phantoms were misclassified as gel wax. This analysis further confirmed the significance of attenuation spectra-based classification of samples.

## 4 Discussion and Conclusion

Commercially available OCT systems for intravascular applications are limited in identifying lipid-containing tissues based on absorption features since the absorption coefficient of lipids in the 1300 nm window is small and relatively featureless. Here, we developed a 1.2  μm swept laser source-based OCT system where the lipid has a higher absorption coefficient. We also demonstrated a post-processing method to simultaneously generate high-resolution OCT intensity images and spectroscopic information of the sample. Spectroscopic information was extracted as wavelength-resolved attenuation spectra, by a single-scattering model. Absorption spectra could be extracted and were in quantitative agreement with published reference data. This combined analysis of spectral and spatial information in OCT scan is a promising method for identifying lipid-containing samples. An OPLS-DA model, calibrated on samples with lipid-like and water-like absorption features, was able to correctly classify 19/20 validation samples. We also implemented an OPLS-DA model based on normal attenuation coefficients and our results indicate that the classification based on normal attenuation coefficients is insignificant.

Although the results are encouraging, our studies were limited to weakly scattering homogeneous samples. Heterogeneity will be challenging in the incoherent averaging step we applied. Incoherent averaging is necessary to reduce the impact of speckle, which is needed for a reliable attenuation fit. Direct extraction of spectral data from the windowed inverse Fourier transform of the full-spectrum A-line is similarly affected by speckle, and incoherent averaging of the spectra would be necessary in that approach.

The lipid absorption coefficient at 1210 nm is $0.175\;{\mathrm{mm}}^{-1}$, whereas the scattering coefficient of common vascular tissues is at least an order of magnitude larger than the absorption coefficient in this wavelength range. Previous studies on attenuation imaging of coronary arteries identified a lipid rich necrotic core have an attenuation coefficient $>10\;{\mathrm{mm}}^{-1}$, which is measured at 1300 nm window where the contribution of scattering is dominating to attenuation coefficient. Within the operating bandwidth of our laser source also expecting a similar range of attenuation coefficient for lipid rich tissues where the contribution of absorption is ∼50 times smaller than that of scattering. In such strongly scattering tissues, the absorption coefficient will be a proportionally smaller component of the overall attenuation, and will thus be more challenging to extract reliably. This would likely also complicate the classification using the OPLS-DA model.

In summary, we demonstrated a 1200 nm SOCT that estimates the wavelength-dependent absorption coefficients of lipid and water containing weakly scattering phantoms. Furthermore, we fitted an OPLS-DA model that was able to discriminate the attenuation spectra of lipid-containing phantoms from water-based phantoms with good predictive ability.
